# Renal function during long-term lithium treatment: a cross-sectional and longitudinal study

**DOI:** 10.1186/s12916-014-0249-4

**Published:** 2015-01-21

**Authors:** Alberto Bocchetta, Raffaella Ardau, Tiziana Fanni, Claudia Sardu, Doloretta Piras, Antonello Pani, Maria Del Zompo

**Affiliations:** Section of Neuroscience and Clinical Pharmacology, Department of Biomedical Sciences, University of Cagliari, Via Ospedale 54, Cagliari, 09124 Italy; Unit of Clinical Pharmacology, Azienda Ospedaliero-Universitaria di Cagliari, Via Ospedale 54, Cagliari, 09124 Italy; Section of Obstetrics, Gynecology and Pediatrics, Department of Surgical Sciences, University of Cagliari, Pediatric Hospital, Via Jenner 1, Cagliari, 09121 Italy; Department of Public Health, Clinical and Molecular Medicine, University of Cagliari, Cittadella Universitaria, Strada Provinciale Monserrato-Sestu Km 0.7, Monserrato, CA 09042 Italy; Nephrology, Dialysis and Transplantation Unit, ‘Giuseppe Brotzu’ Hospital, Piazzale Ricchi 1, Cagliari, 09134 Italy

**Keywords:** Chronic kidney disease, Glomerular filtration, Lithium treatment

## Abstract

**Background:**

The effects of lithium treatment on renal function have been previously shown, albeit with discrepancies regarding their relevance. In this study, we examined glomerular filtration rate in patients treated with lithium for up to 33 years.

**Methods:**

All lithium patients registered from 1980 to 2012 at a Lithium Clinic were screened. Estimated glomerular filtration rate (eGFR) was calculated from serum creatinine concentration using the Modification of Diet in Renal Disease Study Group equation. A cross-sectional evaluation of the last available eGFR of 953 patients was carried out using multivariate regression analysis for gender, current age, and duration of lithium treatment. Survival analysis was subsequently applied to calculate the time on lithium needed to enter the eGFR ranges 45 to 59 mL/min/1.73 m^2^ (G3a) or 30 to 44 mL/min/1.73 m^2^ (G3b). Finally, 4-year follow-up of eGFR was examined in subgroups of patients who, after reduction to an eGFR lower than 45 mL/min/1.73 m^2^ either i) continued lithium at the same therapeutic range or ii) discontinued lithium or continued at concentrations below the therapeutic range (0.5 mmol/L).

**Results:**

In the cross-sectional evaluation, eGFR was found to be lower in women (by 3.47 mL/min/1.73 m^2^), in older patients (0.73 mL/min/1.73 m^2^ per year of age), and in patients with longer lithium treatment (0.73 mL/min/1.73 m^2^ per year). Half of the patients treated for longer than 20 years had an eGFR lower than 60 mL/min/1.73 m^2^. The median time on lithium taken to enter G3a or G3b was 25 years (95% CI, 23.2–26.9) and 31 years (95% CI, 26.6–35.4), respectively. Progression of renal failure throughout the 4-year follow-up after a reduction to an eGFR lower than 45 mL/min/1.73 m^2^ did not differ between the subgroup who continued lithium as before and the subgroup who either discontinued lithium or continued at concentrations below the therapeutic range.

**Conclusions:**

Duration of lithium treatment is to be added to advancing age as a risk factor for reduced glomerular filtration rate. However, renal dysfunction tends to appear after decades of treatment and to progress slowly and irrespective of lithium continuation.

## Background

Lithium is the most effective long-term therapy for bipolar disorder [1,2] and is also effective in unipolar depression [3]. However, some concerns have frequently been raised about its safety [4]. In an article published in 2013 in *BMC Medicine*, Emanuel Severus and Michael Bauer discussed the implications of the risk of lithium-induced nephropathy in long-term treatment [5]. These comments were prompted by the publication of a study by our group regarding 139 patients at different stages of lithium treatment and 70 patients treated with other mood stabilizers. Although based on a cross-sectional evaluation, data indicated a positive correlation between duration of lithium treatment and reduced estimated glomerular filtration rate (eGFR) [6].

In the present study, we extended the cross-sectional analysis to the entire cohort of patients registered at our Lithium Clinic between 1980 and 2012 (n = 1,862). We calculated the prevalence of reduced eGFR by age, sex, and duration of lithium treatment. Moreover, we introduced a longitudinal view in order to calculate the time taken before reduction to an eGFR lower than 60 mL/min/1.73 m^2^ while on lithium and to follow the progression of renal failure in case of continuation or discontinuation of lithium.

## Methods

All lithium patients registered between January 1, 1980, and December 31, 2012, at the Unit of Clinical Pharmacology, University Hospital of Cagliari, were screened. The study protocol was approved by the local Ethics Committee of the University Hospital of Cagliari, Italy (reference number 16/09/CE). All patients gave written informed consent before inclusion in the study. The study was performed in accordance with the Declaration of Helsinki.

To ascertain actual exposure to lithium, duration of treatment was calculated for each patient by adding up the years during which lithium was regularly found within the therapeutic range (0.5 to 1.0 mmol/L) and for whom the interval between checks on serum lithium concentrations at our Unit did not exceed four months.

Serum creatinine concentrations were taken from the panel of laboratory tests requested on an annual basis. The traditional standardization method for serum creatinine was used. The eGFR was calculated from serum creatinine values using the equation proposed by the Modification of Diet in Renal Disease Study Group [7,8], with the ‘186’ correction factor, which also takes into account age, sex, and ethnicity. The following categories of eGFR were considered: higher than 90 mL/min/1.73 m^2^ (G1); 60 to 89 mL/min/1.73 m^2^ (G2); 45 to 59 mL/min/1.73 m^2^ (G3a); 30 to 44 mL/min/1.73 m^2^ (G3b); 15 to 29 mL/min/1.73 m^2^ (G4); and lower than 15 mL/min/1.73 m^2^ (G5). The abbreviations and ranges recall those used by the Kidney Disease Improving Global Outcomes 2012 Clinical Practice Guidelines for the Evaluation and Management of Chronic Kidney Disease (CKD) [9], but it must be noted that these stages are also based on albuminuria categories, which were not included in the present study. To avoid misclassification based on single eGFR determinations, a best-estimate was established based on the last two available values. When the last two eGFR values oscillated between two contiguous ranges, the patient was assigned to intermediate categories (for example G1/G2 or G2/G3a).

To corroborate the results from our previous cross-sectional study regarding a subsample of 139 lithium patients [6], multivariate regression analysis was applied to cross-sectional data regarding the entire cohort of patients. In this case, the last available eGFR was included as the dependent variable. The independent variables were age, sex, and duration of lithium treatment.

Survival analysis was applied to estimate the number of years on lithium required to enter the eGFR ranges 45 to 59 mL/min/1.73 m^2^ (G3a) or 30 to 44 mL/min/1.73 m^2^ (G3b). The median time to reach the different eGFR stages was calculated, in the whole sample, using the Kaplan-Meier method. According to this method, in cases when the stage under consideration had not been reached, data were censored at the last follow-up visit. Kaplan-Meier curves were generated for the entire sample or after stratification into age groups. In the latter case, the log rank test was applied to compare survival distributions. Cox proportional hazards regression was applied to control for age and sex.

The progression of renal failure after a reduction to an eGFR lower than 45 mL/min/1.73 m^2^ was studied in the following two subgroups: i) patients who continued lithium at the same therapeutic range and ii) patients who either discontinued lithium or continued at concentrations below the therapeutic range (0.5 mmol/L). The latter subgroup was combined to allow an adequate sample size, as patients tended to be lost to follow-up at the lithium clinic. Parametric and non-parametric tests were used to compare eGFR values between the two subgroups and between different follow-up intervals.

## Results

Of 1,862 patients in the lithium register, 953 (596 females, 357 males) met the following criteria and were studied: i) having been treated at the lithium clinic for at least one year and ii) having at least one serum creatinine value available to calculate eGFR.

### Cross-sectional data

**Figure 1:**
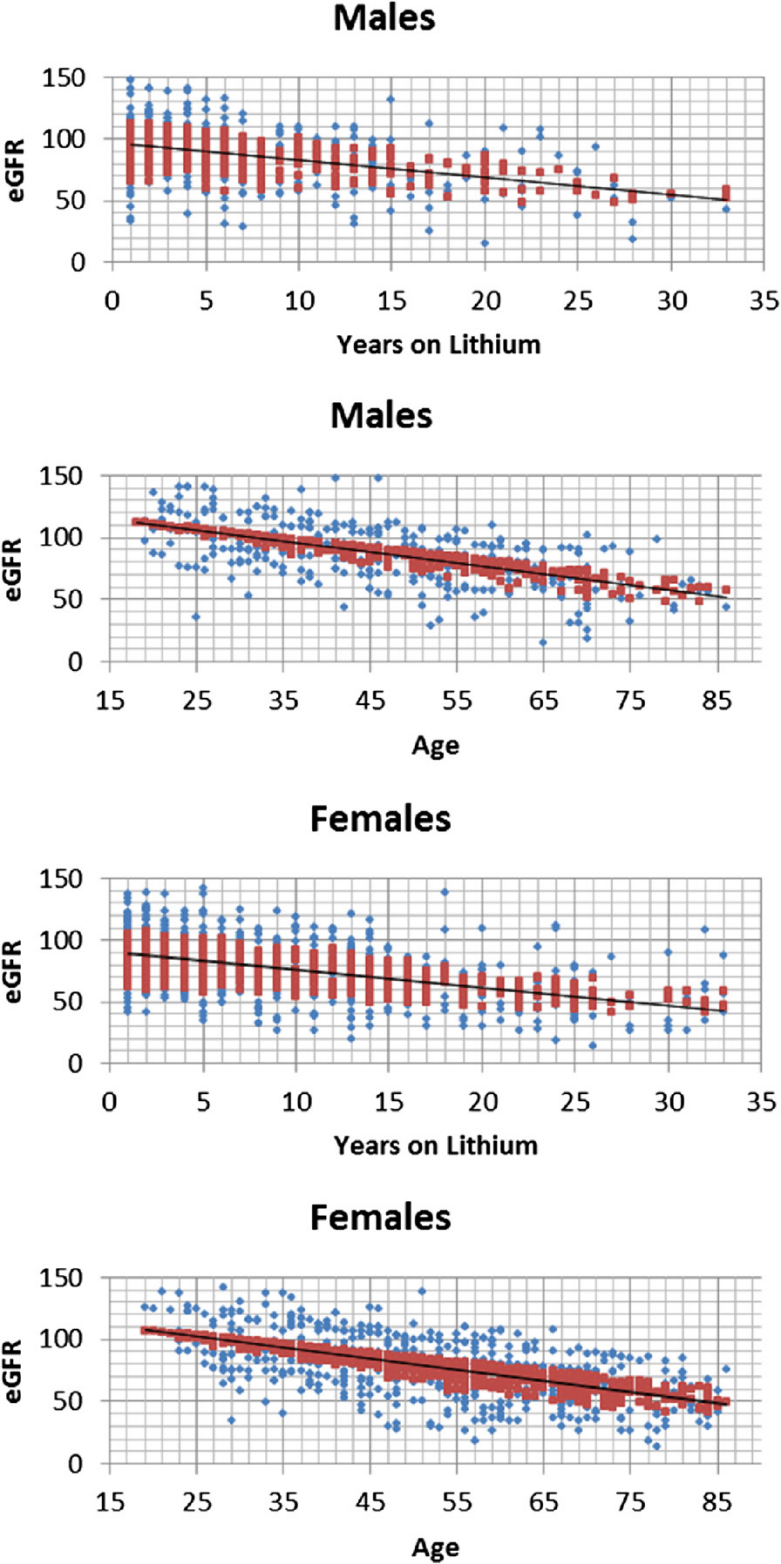
**Last available estimated glomerular filtration rate by current age and duration of lithium treatment in 953 patients.**

Figure 1 and Table 1 show the results from multivariate analysis of the last available eGFR of the 953 patients studied. The results can be summarized as follows: eGFR was lower in women (by 3.47 mL/min/1.73 m^2^), in older patients (0.73 mL/min/1.73 m^2^ per year of age), and in patients with longer lithium treatment (0.73 mL/min/1.73 m^2^ for each year).

**Table 1:** **Multivariate analysis of the last available estimated glomerular filtration rate in 953 patients treated with lithium for up to 33 years**

**Variable**	**Beta**	***P*** **value**	**95% confidence interval**	
Gender (male versus female)	3.47	0.008	0.91	6.02
Current age	–0.73	3.0 × 10^–56^	–0.82	–0.65
Duration of lithium treatment	–0.73	1.8 × 10^–14^	–0.91	–0.55

**Figure 2:**
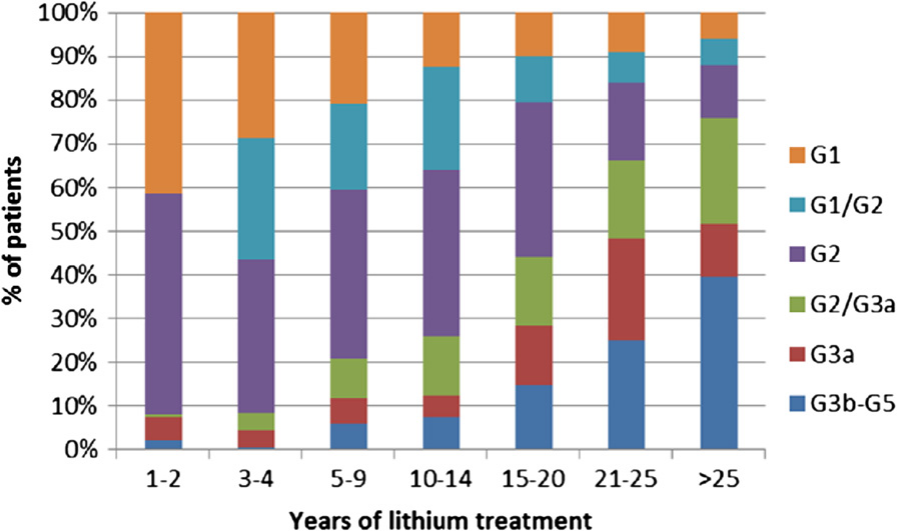
**Prevalence of patients falling into different estimated glomerular filtration rate (eGFR) categories among subgroups exposed to lithium for different times.** The last two available eGFR values were used to assign patients to the following eGFR categories: G1, both values higher than 89 mL/min/1.73 m^2^; G1/G2, one value within G1 and one within G2; G2, both values between 60 and 89 mL/min/1.73 m^2^; G2/G3a, one value within G2 and one within G3a; G3a, both values between 45 and 59 mL/min/1.73 m^2^; G3b–G5, both values lower than 44 mL/min/1.73 m^2^.

Figure 2 shows the proportion of patients falling into various categories of eGFR among subgroups exposed to lithium for different times, just to provide a view of the relevance of reduced eGFR. In this case, the last two available eGFR values were used for cross-sectional evaluation of the current eGFR range to avoid misclassification based on single eGFR determinations.

Although the effect of age is not included, Figure 2 provides a perspective on critical cases. For example, it is noteworthy that, irrespective of age, half of the patients treated with lithium for longer than 20 years had an eGFR lower than 60 mL/min/1.73 m^2^, which usually recommends consultation with a nephrologist, whereas 40% of patients treated for longer than 25 years had an eGFR lower than 45 mL/min/1.73 m^2^, which is often considered a point of no return.

### Longitudinal data

**Figure 3:**
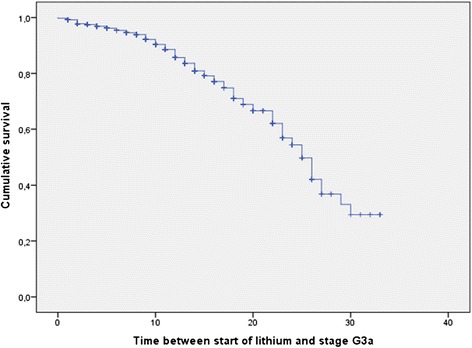
**Kaplan-Meier curve showing years on lithium treatment taken to enter stage G3a (45 to 59 mL/min/1.73 m** ^**2**^ **; 136 events out of 953 patients; 1- to 33-year follow-up).**

**Figure 4:**
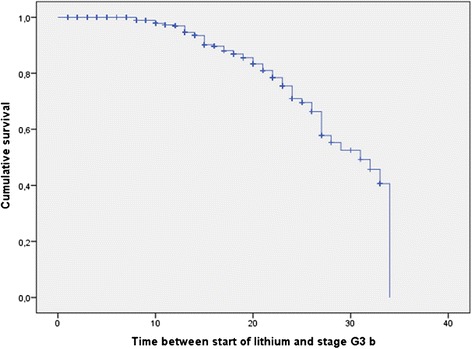
**Kaplan-Meier curve showing years on lithium treatment taken to enter stage G3b (30 to 44 mL/min/1.73 m** ^**2**^ **; 68 events out of 953 patients; 1- to 33-year follow-up).**

Figures 3 and 4 show the Kaplan-Meier curves regarding the entire sample. Of the 953 patients studied, 136 declined to an eGFR lower than 60 mL/min/1.73 m^2^ and 68 to an eGFR lower than 45 mL/min/1.73 m^2^; the median time taken was 25 years (95% confidence interval (CI), 23.2–26.9) and 31 years (95% CI, 26.6–35.4), respectively. The median time taken to decline from range G3a to G3b was 8 years (95% CI, 6.6–9.4).

Stratification into age subgroups (data not shown) revealed no differences regarding the time required to enter the G3b range, whereas the time to enter the G3a range differed significantly (overall log rank test,15.4; *P* <0.0001). In this case, median time was not reached and could not be calculated in the age subgroup younger than 40 years, whereas it was shorter in the age subgroup ≥60 years (23 years; 95% CI, 19.7–26.3) as compared to the age subgroup 40–59 years (26 years; 95% CI, 24.5–27.5).

Cox regression revealed the following age and sex effects: i) the hazard ratio (HR) to enter range G3a increased with age (HR, 1.03; 95% CI, 1.02–1.05); ii) men had a lower risk of entering range G3b as compared to women (HR, 0.49; 95% CI, 0.25–0.96).

Table 2 shows the outcome in the subgroups of patients who had a reduction in eGFR to lower than 45 mL/min/1.73 m^2^ (set as time zero) and i) continued lithium at the usual therapeutic concentration range (0.50 to 1.0 mmol/L) or ii) either discontinued lithium or continued at concentrations below the therapeutic range (0.5 mmol/L).

**Table 2:** **Follow-up of estimated glomerular filtration (eGFR) in subgroups of patients with a diagnosis of renal failure, continuing lithium treatment as before or discontinuing/reducing lithium intake**

**Follow-up interval after the diagnosis of renal failure (years)**	**Patients who continued lithium as before**		**Patients who discontinued or reduced lithium below the therapeutic range**	
	**Mean eGFR**	**n**	**Mean eGFR**	**n**
0	39.3	54	37.6	45
1	41.1	39	39.5	28
2	38.6	30	37.9	23
3	36.7	22	33.8	18
4	36.2	24	32.1	15

There were no significant differences between subgroups or between year 1 to 4 and year zero (unpaired tests).

The two subgroups did not differ from one another with regard to mean eGFR at time zero. No significant between-group differences were evident in eGFR up to 4-year follow-up. The median decline in eGFR did not differ from one subgroup to another (*P* = 0.305 at Mann-Whitney *U* test).

## Discussion

Our results corroborate previous findings indicating that the duration of lithium treatment must be added to advancing age as a risk factor for reduced renal function in patients with recurrent or chronic affective illness. The longitudinal part of this study, including a large cohort of patients treated at a specialized facility for up to 33 years, provides suggestions that can help clinicians make decisions regarding long-term lithium treatment.

In the cross-sectional multivariate analysis of the last eGFR from the entire sample, we found that older patients had lower values (0.73 mL/min/1.73 m^2^ per year of age). This result is consistent with the eGFR decline recently reported in a longitudinal analysis of the general population from a Sardinian region (0.79 mL/min/1.73 m^2^ per year over an average seven-year follow-up) [10].

The cross-sectional analysis from this study revealed that, after age and sex corrections, there is an additional negative effect of the duration of lithium treatment (0.73 mL/min/1.73 m^2^ per year of treatment). Therefore, we can estimate that long-term lithium exposure to therapeutic concentrations may double the eGFR decline associated with advancing age.

However, according to our longitudinal evaluation, it appears that it may take decades before patients fall into severely reduced eGFR ranges. This is perhaps one of the principal reasons why early studies with limited follow-up had not pointed out the relevance of reduced glomerular function in lithium-treated patients. Indeed, a recent systematic review and meta-analysis of lithium toxicity profile concluded that “*there is little evidence for a clinically significant reduction in renal function in most patients, and the risk of end-stage renal failure is low*” [4]; the conclusions, however, were based on prospective studies with a mean observation time of one year on lithium. Further, the meta-analysis does not include data from naturalistic studies conducted under ordinary clinical conditions and is not equipped to assess the epidemiological relevance of lithium-related renal impairment, as stated by Müller-Oerlinghausen *et al.* in *BMC Medicine* [11].

Nevertheless, adverse renal effects of lithium have long been known, varying from very frequent reversible polyuria [12] to irreversible kidney damage [13,14]. Early studies reported tubular damage but no (or minimal) glomerular damage (for a review, see [15]). Analysis of studies published from 1979 to 1986 comprising 1,172 patients concluded that glomerular filtration rate was normal in 85% of unselected patients on chronic lithium therapy and that the remaining 15% of patients displayed only a mild reduction in glomerular filtration rate, clustering at approximately 60 mL/min [16]. A more recent study by Tredjet *et al.* [17] reported a prevalence of 34.4% with lower than 60 mL/min /1.73 m^2^ [17]. Up to the 1990’s, severe renal failure and end-stage renal disease (ESRD) were considered unlikely events [18]. Subsequent studies principally originated from nephrology facilities and focused on the small number of extremely severe cases [19-21]. For example, Markowitz *et al.* [19] studied 24 patients with renal biopsy and concluded that: i) lithium may be responsible for combined glomerular and tubular-interstitial damage; ii) toxicity may be irreversible, even despite lithium withdrawal; and iii) early detection is essential to prevent progression to ESRD. Our results are comparable to those from Presne *et al.* [20] who, based on data from 74 patients, concluded that lithium-induced CKD progresses slowly, its rate of progression related to the duration of exposure. Bendz *et al.* [21] described 32 cases in Sweden of ESRD associated with long-term lithium exposure, underscoring the potential role of the relatively higher therapeutic ranges adopted in the 1960 to 1970’s as compared to current guidelines. This may not be the case with our lithium cohort, dating no further back than 1980 and maintained within a therapeutic range between 0.5 and 1.0 mmol/L. We have planned to investigate the effect of lithium concentration in another account.

The lack of large surveys has recently prompted studies addressing the epidemiological relevance of less severe CKD stages, compared to studies from nephrology facilities. An advance in epidemiological surveys of large populations was represented by the demonstrated reliability of eGFR calculated from routine laboratory determinations of serum creatinine [7,8]. Previous studies of renal dysfunction in lithium patients had often relied on serum creatinine values alone, setting the alarm signal at 140 mmol/L (1.6 mg/dL), which might have resulted in an underestimated prevalence of renal failure, especially among older patients [18].

More recent studies using eGFR changed the perspective. For example, Bassilios *et al.* [22] found a very high prevalence of eGFR lower than 60 mL/min/1.73 m^2^ among 695 outpatients referred to their private laboratory in Paris for measurement of lithium serum concentrations. Reduced eGFR was especially prevalent in older age groups (78 to 86% of patients older than 60 years had an eGFR lower than 60 mL/min/1.73 m^2^), consistent with the effect of age found in our study. However, unlike our study, theirs did not analyze duration of lithium treatment, and the majority of patients were no longer being treated at specialized lithium clinics [22]. As stated by Müller-Oerlinghausen *et al*. [11], “*regular kidney function monitoring is often lacking in practice*”, and the “*French study shockingly showed that creatinine serum levels had not been performed in 40%*” of lithium patients referred to their laboratory; the comment also underlined that, unfortunately, the risk of renal dysfunction “*might not be that rare, even in subjects properly managed on lithium*”. Prevalence rates were somehow lower among patients from our cohort who were regularly treated at a lithium clinic as compared to those from the French survey, but, in any case, rates can be considered impressive as compared to the 15% prevalence calculated by the review of earlier studies of unselected patients on chronic lithium [16].

In the debate regarding the epidemiological relevance of renal dysfunction among lithium-treated patients, our results can be added to those from another recent retrospective cohort study examining the risk of renal impairment (CKD stage 3) or failure (CKD stages 4 or 5) among 6,360 patients with bipolar disorder, treated or not with lithium and recorded from 1990 to 2007 in the UK General Practice Research Database [23]. In that UK study, lithium use (defined as at least one prescription of at least 30 days duration) was associated with a HR for renal failure of 2.5 and a HR for renal impairment of 2.7. Risk particularly increased among the 50-or-older age group.

The principal results from both the UK study and our own are similar. One advantage of our study is that lithium exposure was checked with regular serum monitoring. Moreover, duration of treatment and actual eGFR measurements were included in our analysis and we were able to longitudinally describe the relatively slow eGFR decline.

On the other hand, our study has obvious limitations. For example, we have not explored the role of known renal risk factors (diabetes, hypertension, use of NSAIDs, smoking, etc.), and there was no comparison with a group of patients suffering from recurrent mood disorder but not exposed to lithium.

We maintain that naturalistic studies from lithium clinics may be relevant with regard to the risk of renal failure. As stated by Müller-Oerlinghausen *et al.* [11], “*when to discontinue lithium because of serious renal problems is a particularly vexing problem. This decision cannot be made solely by the treating nephrologist, but also requires expert psychiatric evaluation of the benefits and true risks that the individual patient can expect from his/her lithium medication in future years*”. Indeed, the benefits obtained from lithium must be regarded not only in terms of stabilization of recurrent mood disorders, but also in normalization of their otherwise increased mortality, mainly due to suicide [24-26]. Werneke *et al.* [27] conducted a decision analysis simulating the decision process between physicians and patients. The analysis addressed two questions: ‘Should lithium be recommended at the beginning of treatment in view of a small but significant risk of ESRD later in life?’ and ‘Should lithium continuation be recommended even in the presence of long-term adverse renal effects?’

Surprisingly, guidelines regarding such important questions are often based on limited literature data – the aim of this study was to aid in filling this gap. To our knowledge, this is the first attempt to actually estimate the time needed to enter the eGFR ranges generally considered crucial in the management of lithium treatment. With regard to the outcome of renal failure in lithium-treated patients, data from the literature are particularly scarce and controversial. A few studies suggest that advanced stages of renal failure appear to progress irrespective of the continuation or discontinuation of lithium treatment, while others suggest that discontinuation may be beneficial [19-21,28]. Our follow-up data add a small contribution to the debate, suggesting that the reduction in eGFR is slow, even after falling into eGFR values lower than 45 mL/min/1.73 m^2^ and irrespective of lithium discontinuation or reduction below the usual therapeutic range.

Opting for discontinuation must be cautious considering previous evidence that mortality may be disproportionally high in patients with irregular attendance at our lithium clinic. For example, 43 suicides were recorded from 1980 to 2002 among patients in our lithium register: 42 of 43 were not under regular lithium prophylaxis; on the other hand, renal failure was recorded as the main cause of death in only two cases [26].

## Conclusions

These results confirm that reduction in renal function, even if rarely progressing to end-stage renal failure, should be reconsidered in the debate on the lithium toxicity profile. Duration of lithium treatment is to be added to advancing age as a risk factor. However, the risk of renal dysfunction must be weighed against the protective effects of lithium on the recurrence of mood disorders, quality of life, and suicide. As continuation or discontinuation of lithium does not appear to significantly influence the progression to ESRD once kidneys are already damaged, it would be desirable that future studies identify early markers of lithium nephrotoxicity.

## Abbreviations

CI: Confidence interval
CKD: Chronic kidney disease
eGFR: Estimated glomerular filtration rate
ESRD: End-stage renal disease
HR: Hazard ratio
